# Oxidative chemical vapor deposition of polyaniline thin films

**DOI:** 10.3762/bjnano.8.128

**Published:** 2017-06-16

**Authors:** Yuriy Y Smolin, Masoud Soroush, Kenneth K S Lau

**Affiliations:** 1 Department of Chemical and Biological Engineering, Drexel University, Philadelphia, PA 19104, USA

**Keywords:** conducting polymers, emeraldine oxidation state, oxidative chemical vapor deposition, polyaniline, thin film processing

## Abstract

Polyaniline (PANI) is synthesized via oxidative chemical vapor deposition (oCVD) using aniline as monomer and antimony pentachloride as oxidant. Microscopy and spectroscopy indicate that oCVD processing conditions influence the PANI film chemistry, oxidation, and doping level. Fourier transform infrared spectroscopy (FTIR), scanning electron microscopy (SEM), and X-ray photoelectron spectroscopy (XPS) indicate that a substrate temperature of 90 °C is needed to minimize the formation of oligomers during polymerization. Lower substrate temperatures, such as 25 °C, lead to a film that mostly includes oligomers. Increasing the oxidant flowrate to nearly match the monomer flowrate favors the deposition of PANI in the emeraldine state, and varying the oxidant flowrate can directly influence the oxidation state of PANI. Changing the reactor pressure from 700 to 35 mTorr does not have a significant effect on the deposited film chemistry, indicating that the oCVD PANI process is not concentration dependent. This work shows that oCVD can be used for depositing PANI and for effectively controlling the chemical state of PANI.

## Introduction

Conducting polymers (CPs) have attracted considerable attention in recent years for their use in solar cells [1–6], batteries [7], supercapacitors [8–12], sensors [13], biosensors [14], and microelectronics [15–16]. As devices continue to decrease in size, the integration of conducting polymers within nanomaterials using conventional solvent-based methods becomes considerably more challenging due to the lack of solubility in common commercial solvents, which limits processability and leads to poor wettability. These challenges can be overcome with oxidative chemical vapor deposition (oCVD). oCVD is a single step, solvent-free polymerization and coating technique, which has previously been used to deposit thin and ultrathin conducting polymer films, including polypyrrole, polythiophene (PTh), and poly(3,4-ethylenedioxythiophene) (PEDOT), without the limitations of solvent-based techniques [17]. The oCVD process provides better control over the deposition (such as film thickness, conformality, uniformity, morphology) than current solution-based techniques such as chemical bath deposition [18], electrodeposition [19], and casting from suspension [20]. As a result, oCVD has garnered significant attention in recent years as an advantageous route for depositing conducting polymer thin films without the need of a solvent or a conductive substrate, which naturally makes the process amenable in a wide range of applications [17,21]. Other methods such as plasma-enhanced CVD (PECVD) have previously been used to make conformal and uniform polymer films. However, the high energies in PECVD of polymers often result in the loss of functionality and degradation of a stoichiometric linear homopolymer [17]. Laser-based techniques, such as pulsed laser deposition (PLD), matrix-assisted pulsed laser evaporation (MAPLE), and laser-induced forward transfer (LIFT), have also been used to deposit polymer thin films [22]. However, these laser based methods also often lead to polymer degradation or a reduction in molecular weight [23–25]. Resonant infrared laser vapor deposition (RIR-LVP) has been used to deposit PEDOT but conductivity and morphology were highly dependent on the solvent matrix and the laser irradiation wavelength, and MAPLE led to a film that was electrically insulating [26].

Previous studies by Gleason and coworkers highlighted oCVD’s advantages in the conformal deposition of PEDOT films with tunable nanoporosity [27], and demonstrated PEDOT as a neutral hole-transporting polymer for enhancing solar cells efficiency and lifetime [28]. oCVD PEDOT was also used to encapsulate flexible organic photovoltaics [29] and in the fabrication of organic photovoltaic circuits on unmodified paper [30]. Likewise, our group demonstrated the utility of oCVD in the synthesis of PTh and showed that the polymer conjugation length and electrical conductivity can be tuned by adjusting the oCVD processing conditions [31]. We further deposited ultrathin (4–6 nm) conformal and uniform PTh coatings within porous nanostructures, including anodized aluminum oxide, mesoporous TiO_2_, and activated carbon; these oCVD PTh coatings resulted in enhanced charge storage due to preservation of the surface area and pore space within the nanostructures [32]. As a result, PTh-coated carbon electrodes showed a 50% increase in specific capacitance and excellent cycle life even after 5000 cycles due to the robust ultrathin coatings [32]. In addition, our study of the copolymerization of thiophene and pyrrole via oCVD showed enhanced conductivity and stability of the copolymers [33]. In view of experimental evidence that oCVD conducting polymers show favorable properties and can be easily processed, and that PANI has many advantages over PEDOT and PTh, including high theoretical capacitance (55% higher than PTh), low monomer cost, better stability, and high electrical conductivity [34–35], the deposition of PANI by oCVD is expected to open up new possibilities for significantly improving the performance and stability of energy storage devices along with other device classes such as sensors.

**Figure 1:**
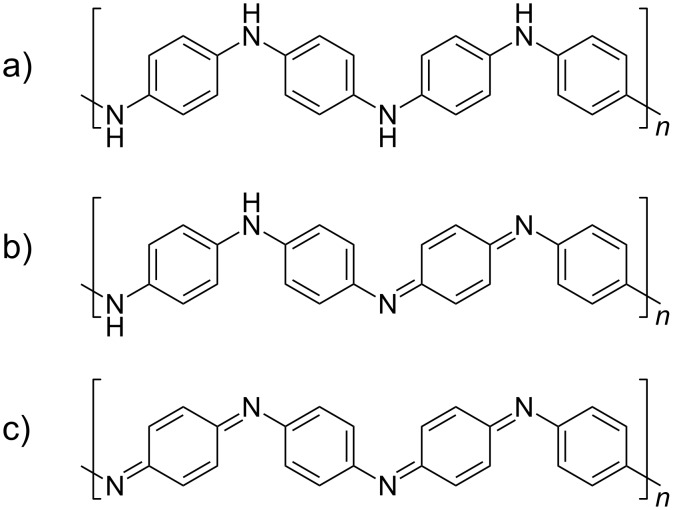
Chemical structure of the primary oxidation states of PANI in the undoped, base form. (a) Fully reduced leucoemeraldine PANI composed of benzenoid groups, (b) emeraldine PANI state composed of a 1:1 ratio of benzenoid and quinoid groups, and (c) fully oxidized pernigraniline PANI composed of quinoid groups.

Therefore, this work aims to demonstrate the synthesis of PANI by the oCVD approach, in particular, to investigate systematically how oCVD processing variables influence PANI thin film deposition and chemistry. This processing knowledge is essential for oCVD PANI applications and for optimizing the performance of devices that use PANI coatings. Figure 1 shows the three basic oxidation states of PANI in the base (undoped) form. The fully reduced leucoemeraldine state, which is colorless, is composed fully of benzenoid groups (Figure 1a). At the other extreme, the fully oxidized pernigraniline state, is composed of all quinoid groups and produces a deep blue or violet color (Figure 1c). In between, the partially oxidized emeraldine form is composed of a 1:1 ratio of benzenoid and quinoid groups, which appears as a vivid green (Figure 1b). This emeraldine state is desired from an electrochemical standpoint, because its electrical conductivity is 10 orders of magnitude greater compared with the other two states [36]. Therefore, this work addresses how oCVD can be operated to tune the deposition and chemistry of emeraldine PANI.

## Experimental

### oCVD deposition of polyaniline

**Figure 2:**
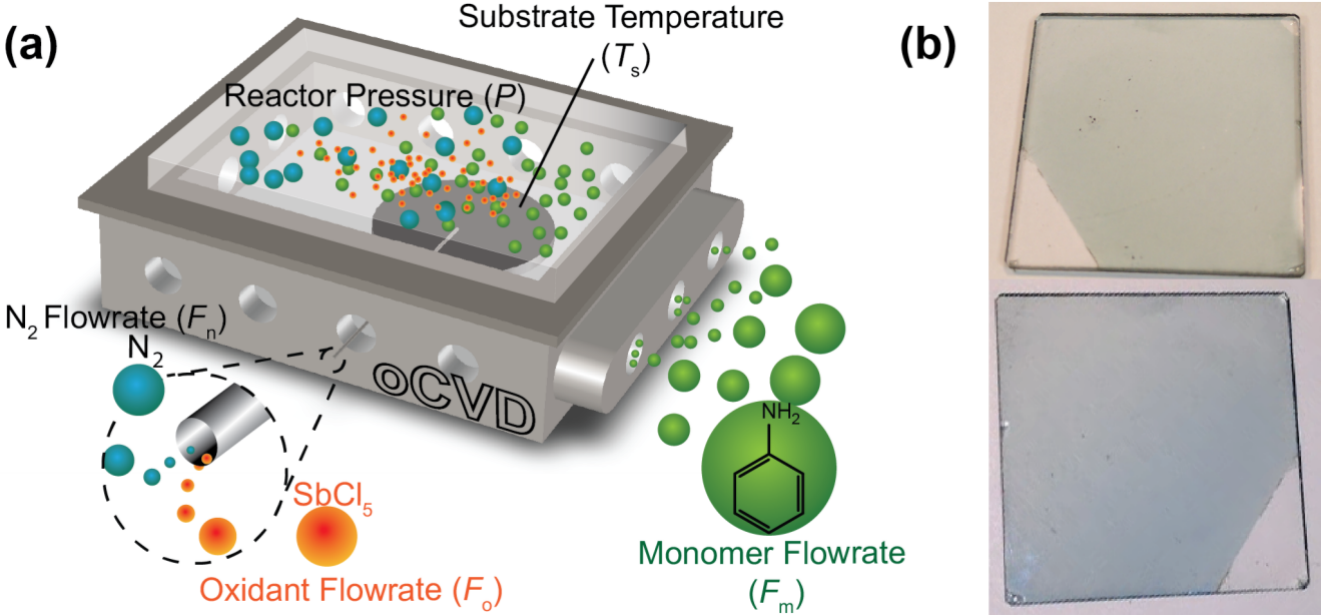
(a) oCVD process highlighting important process parameters, including substrate temperature (*T*_s_), feed flowrates (*F*_o_, *F*_m_, *F*_n_), and reactor pressure (*P*), for the synthesis of PANI. (b) oCVD PANI deposited on quartz glass (1” × 1”) showing the emeraldine (green) and pernigraniline (blue) states. The uncoated portion was masked by tape during deposition.

The oCVD process for PANI (Figure 2a) involves flowing vapors of the monomer (aniline) and the oxidant (antimony pentachloride, SbCl_5_) into the reactor continuously. Nitrogen gas is used as an inert carrier to help transport the oxidant and as a diluent to help control polymerization reactions. The monomer and oxidant are delivered in separate quarter-inch stainless-steel tubes to isolate the reactants prior to entering the reaction chamber and minimize polymerization and blockage in the gas delivery manifold system. Upon entry into the oCVD reaction chamber, the monomer and oxidant vapors adsorb onto the substrate surface and surface polymerize via a step-growth mechanism, which mimics the oxidative chemical polymerization used in solution-based processes to grow conducting polymers [17].

Aniline (Sigma-Aldrich, ACS reagents, >99.5%) and antimony pentachloride oxidant (Sigma-Aldrich, 99%) were used as-received without further purification. Separate source vessels containing antimony pentachloride and aniline were heated to 60 °C to produce sufficient vapors that were metered into the oCVD reaction chamber using low-flow precision metering valves (Swagelok). The “base-case” (BC) deposition conditions were used as a starting point to explore how processing conditions affected film chemistry (Table 1). It had a reactor pressure (*P*) of 700 mTorr, controlled using a downstream throttle value and pressure controller. The monomer and oxidant flowrates (*F*_m_ and *F*_o_, respectively) were set at 1 and 0.8 sccm (standard cm^3^·min^−1^), respectively. Nitrogen gas, maintained at a flowrate (*F*_n_) of 1 sccm by a mass flow controller (MKS 1479A), was also sent through the oxidant line as a diluent. PANI films with a target thickness of 250 nm were deposited on silicon wafers and quartz glass substrates, which were placed on a stage controlled at 90 °C (*T*_s_) using backside contact with a recirculating thermal fluid (distilled H_2_O).

**Table 1:** oCVD process conditions for PANI synthesis and deposition.^a^

Run	*P* (mTorr)	*T*_s_ (°C)	*F*_o_ (sccm)	*F*_m_ (sccm)	*F*_n_ (sccm)	Sample notation

1	700	90	0.80	1	1	BC
2	100	90	0.80	1	1	P1
3	35	90	0.80	1	1	P2
4	700	90	0.30	1	1	F1
5	700	90	0.15	1	1	F2
6	700	25	0.80	1	1	LT-BC
7	700	25	0.30	1	1	LT-F1

^a^*P* = reactor pressure; *T*_s_ = substrate temperature; *F*_o_, *F*_m_, *F*_n_ = flowrates of the oxidant (antimony pentachloride), monomer (aniline), and nitrogen gas, respectively.

oCVD processing conditions were then systematically varied from the base case, according to Table 1, to understand how they affect the resulting polymer film. First, the reactor pressure was varied from the base case of 700 mTorr to 35 mTorr (*P* series: BC, P1, P2). A lower reactor pressure should lead to more conformal deposition and polymerization given the greater mean free path and lower concentration. Second, the oxidant flowrate was varied from the base case of 0.8 sccm to 0.15 sccm (*F* series: BC, F1, F2) to investigate possible changes in the oxidization state and doping level of the PANI film due to the antimony pentachloride oxidant. In addition to the reactor pressure and oxidant flowrate series of runs, two additional conditions were carried out at a lower substrate temperature of 25 °C compared to their high temperature counterparts (LT series: LT-BC, LT-F1). The decrease in the temperature may promote surface adsorption over reaction that can impact polymer growth, conjugation length, and chemistry.

In addtion to examining the as-deposited films, deposited samples were also soaked in tetrahydrofuran (THF >99.9%, Sigma-Aldrich) for 3 h and dried in a vacuum oven at 70 °C for 14 h. The washing process has previously been used to improve film properties, such as conductivity and stability, and often results in a much smoother film surface [17]. Besides THF, methanol is a common solvent that is used in the washing process, and acid-washing (e.g., HCl, HBr, H_2_SO_4_) has also been explored as a way to improve film conductivity by improving chain packing and increased doping [37–38]. The washed films were compared with their as-deposited counterparts to understand how soaking changes the oCVD PANI chemistry. Previous work on other oCVD conducting polymer films have shown that post-deposition rinsing improves film properties such as conductivity and stability by removing residual oxidant, short-chain oligomers, and unreacted monomer [17]. For example, Nejati et al. [31] have shown that washing oCVD PTh films removes the oxidant dopant and soluble portions of the film, which from UV–vis analysis was composed of short chain oligomers of five repeat units or shorter. Work on PEDOT hypothesizes that washing may also lead to tighter chain packing as evident by reduced degradation from water vapor and oxygen exposure [17,37–40]. Therefore, we expect that washing of the oCVD PANI films would remove the antimony pentachloride as well as any soluble oligomeric components that might lead to unfavorable electrochemical properties.

### Thin film characterization

As-deposited and washed PANI films were analyzed by Fourier transform infrared spectroscopy (FTIR), X-ray photoelectron spectroscopy (XPS), and scanning electron microscopy (SEM). FTIR spectra were acquired using a Thermo Nicolet 6700 spectrometer in transmission mode using an MCT/A detector at a resolution of 4 cm^−1^ and averaged over 128 scans. An FTIR spectrum of aniline monomer was also acquired in attenuated total reflectance (ATR) mode. Top-down SEM images were taken using a Zeiss Supra 50VP with the in lens detector at 15 kV and a working distance of 4 mm. The images, acquired using line integration with 7 repeats, were used to estimate film thicknesses. Prior to SEM imaging, samples were sputtered with Pt for 30 s. XPS analysis was conducted using a Physical Electronics VersaProbe 5000 with a micro-focused monochromatic scanned X-ray beam from an Al Kα X-ray source (1486 eV photons) at a spot size of 100 µm, 25 W, and 15 kV. High resolution C1s, N1s, Cl2p, and Sb3d spectra were recorded with a pass energy of 23.5 eV and an energy step of 0.05 eV for a total of 512, 2048, 256, and 256 scans, respectively.

## Results and Discussion

### FTIR of as-deposited oCVD PANI films

Based on the oCVD approach, uniform PANI film depositions were performed on quartz glass substrates, and as seen in Figure 2b, the deposited films can have a vivid green or deep blue color depending on the oCVD conditions. Qualitatively, the colors indicate that PANI in the emeraldine or pernigraniline state, respectively, was formed. To better understand how PANI film chemistry and properties can be influenced by oCVD deposition conditions, a series of deposition runs that systematically looked at some of the critical oCVD processing variables were carried out (Table 1).

**Figure 3:**
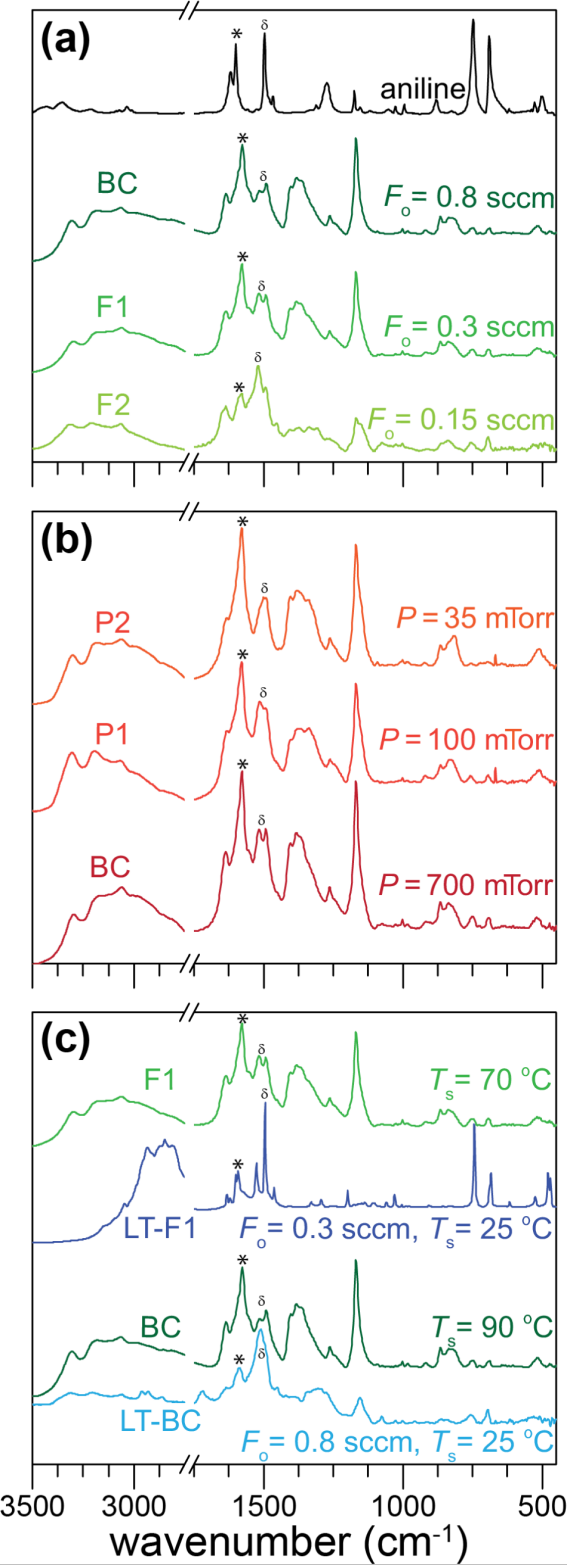
FTIR of as-deposited oCVD PANI films based on the experimental conditions in Table 1. Effect of (a) reactor pressure, (b) oxidant flow rate, and (c) substrate temperature on oCVD PANI chemistry. The quinoid and benzenoid groups are labeled by * and δ, respectively.

For the base case BC, as seen in Figure 3a (0.8 sccm in the *F* series) or Figure 3b (700 mTorr in the *P* series), the FTIR spectrum has peaks that are indicative of the salt form of PANI (doped form, see XPS results below), suggesting that the oxidant dopes the PANI film that is formed. This simultaneous polymerization and doping has been observed previously, for example, with the deposition of oCVD PTh using vanadium oxytrichloride as the oxidant [31]. As discussed in our previous oCVD PANI work [41], the polymerization and doping of polyaniline using oCVD are essentially analogous to chemical oxidative polymerization and acid doping using liquid processing. In the presence of an oxidizing agent, polymerization is believed to proceed via the formation of cation radicals and the electrophilic attack of aniline monomer [42], while in tandem the polymer can be p-doped and charged-balanced with a counterion dopant like chloride [43]. The PANI characteristic peaks are located at 3304, 3064, 1577, 1490, 1382, 1168, 821, and 516 cm^−1^. The 3304 and 3000–3100 cm^−1^ peaks are assigned to NH and CH stretching, respectively, on the aromatic ring of PANI [44–46]. The 1168 cm^−1^ peak is attributed to –NH^+^= stretching and in-plane CH vibrations that suggests the formation of PANI in the salt (doped) form [46–47]. The 821 cm^−1^ peak is typically assigned to out-of-plane CH vibrations [47] that is consistent with high molecular weight PANI due to para-di-substitutions and confirms para-coupling of the constitutive aniline units [48–49]. The quinoid and benzenoid peaks are at 1577 and 1490 cm^−1^, respectively [50], while the 1382 cm^−1^ peak is specifically CN stretching in the quinoid region of the film. The presence of benzenoid and quinoid peaks implies that both amine (N–C) and imine (N=C) units exist within the polymer chains. From the ratio of the 1577 to 1490 cm^−1^ peak intensities, it is possible to determine the oxidation state of the film [51]. For the as-deposited BC film, the ratio of the peak intensities is 1.77, which suggests that the deposited film is mostly composed of quinoid groups and close to the fully oxidized pernigraniline state, as also shown by the blue color of the film (Figure 2b).

For the *F* series, as the oxidant flowrate (*F*_o_) decreases, there is not a significant change in the FTIR spectra of oCVD PANI until a flowrate of 0.15 sccm is used (Figure 3a). First, the peak at 1579 cm^−1^ dramatically decreased from the higher *F*_o_ conditions. This signifies that there is less quinoid groups in the film and therefore the film is much less oxidized. This is expected because the oxidant flowrate is more than 5 times lower than the base case and therefore much less oxidant is available for oxidizing the PANI film. Second, the peaks of the lowest *F*_o_ condition (F2) also indicate that some of the film may contain oligomers. For instance, the peak at 1635 cm^−1^ can be assigned to NH scissoring vibrations of the aromatic amines [52] characteristic of an oligomeric structure. Also, the peak at 1382 cm^−1^, which is the CN stretch in the quinoid structure, becomes nearly indistinguishable and so further confirms that the F2 film contains less quinoid rings. In contrast, the peaks at 749 and 688 cm^−1^, which correspond to CH out-of-plane bending and out-of-plane ring deformation, respectively, of mono-substituted phenylene rings [48] increase in intensity. These peaks are associated with oligomers of around four repeat units, and indicate that a portion of the film is likely composed of oligomers. In fact, if one looks at the monomer spectrum in Figure 3a, these peaks at 749 and 688 cm^−1^ are strong and very sharp. Also, the broadening of the quinoid and benzenoid ring bands signifies a larger distribution of various quinoid structures, which has been reported for aniline oligomers [53]. Therefore, for the deposition of PANI by oCVD, a sufficiently high oxidant/monomer flowrate ratio (>0.3 for the conditions studied here) is required to deposit a film with more oxidized and higher molecular weight PANI while a lower ratio leads to a film that likely contains soluble oligomeric components.

For the *P* series, as the reactor pressure (*P*) decreases from 700 to 35 mTorr (Figure 3b) while maintaining a sufficient oxidant concentration, there is minimal change in the FTIR spectra. This indicates that the PANI chemistry is not very sensitive to pressure variations, at least when there is enough oxidant. Furthermore, with the deposition time held constant (5 min), the deposited film thickness and therefore the deposition kinetics did not change with pressure. Typically, lower reactor pressures would lead to slower kinetics. However, this does not seem to be the case for the oCVD parameter space studied here. Therefore, our conjecture is that the oCVD PANI process is not sensitive to reactant concentrations under these deposition conditions, and that the monomer and oxidant are most likely in excess to have any influence on deposition behavior.

For the runs in which substrate temperature (*T*_s_) was varied, their FTIR spectra can be compared, as shown in Figure 3c. For the LT-BC condition at 25 °C compared to BC at 90 °C (with both at the higher 0.8 sccm oxidant flow), there are several changes. First, the intensity of the 1580 cm^−1^ peak decreases, signifying proportionally fewer quinoid groups in the film and a lower oxidation state. Also, the peak at 1382 cm^−1^, assigned to CN stretching vibration in the quinoid region, is smaller, which further confirms that the LT-BC film contains a smaller amount of quinoid groups. Previous work on oCVD PEDOT showed similar trends with a lower stage temperature yielding lower conjugation length and dopant incorporation [54–55]. Interestingly, the LT-BC spectrum (25 °C, 0.8 sccm oxidant) is very similar to that of F2 (90 °C, 0.15 sccm oxidant), which is believed to have a lower oxidation state and an appreciable amount of oligomers. This suggests that a low substrate temperature has an equivalent effect to reducing the amount of oxidant, which may be the result of more favorable adsorption of short chain oligomers at lower temperatures or slower kinetics at the surface. Further, for the LT-F1 condition, which is now at the low temperature of 25 °C as well as a lower oxidant flow of 0.3 sccm, the film loses most of the FTIR peaks associated with long chain PANI and appears to consist mostly of oligomers, which is supported by the peaks located at 1600, 1525, 1495, 1198, 1030, 684, and 743 cm^−1^. Previous studies of aniline oligomers revealed that the aromatic ring peaks from 1590 to 1510 cm^−1^ are extremely sensitive to the oligomer chain structure and the relative intensity of the 1600 to 1525–1495 cm^−1^ peaks decreases with fewer quinoid rings in the chain [53]. Therefore, the LT-F1 film likely does not have many quinoid structures. Furthermore, the same work showed that oligomeric films lead to ≈10 cm^−1^ shift to higher wavenumbers for the benzenoid and quinoid peaks. Comparing LT-F1 to F1, we see a 5 and 23 cm^−1^ shift to higher wavenumbers for the benzenoid and quinoid peaks, supporting the hypothesis that a predominantly oligomeric film is formed. In fact, the LT-F1 spectrum is very similar to oligomers that are 2–3 aniline repeat units long [53]. This is also why the LT-F1 film is very similar to aniline monomer, although it is unlikely that the film contains any pure aniline since the monomer is sufficiently volatile under vacuum and most likely pumped out after lowering the reactor pressure to base pressure at the end of the deposition run.

### FTIR of washed oCVD PANI films

**Figure 4:**
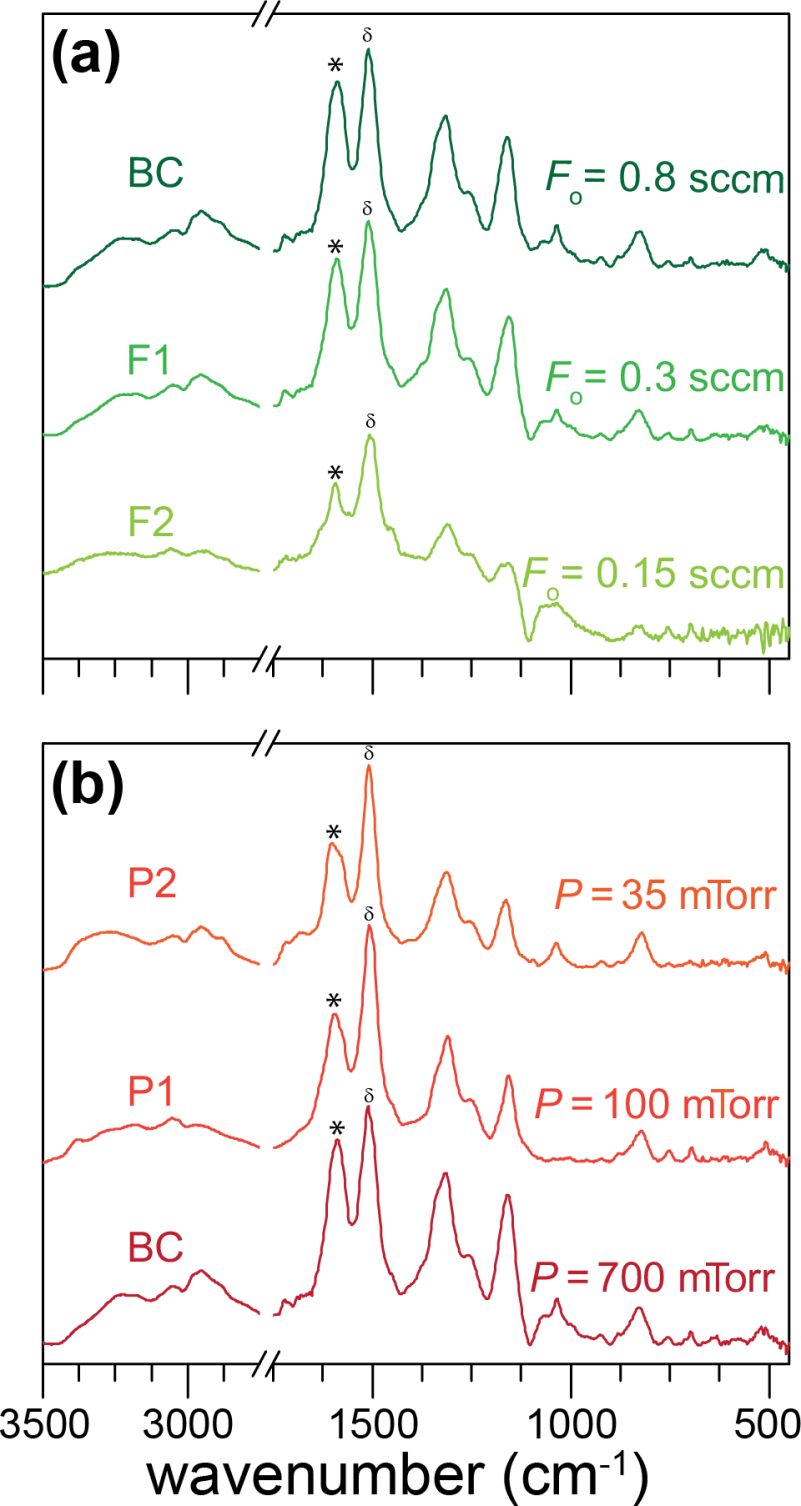
FTIR of washed oCVD PANI films based on the experimental conditions in Table 1. Effect of (a) reactor pressure, and (b) oxidant flow rate on oCVD PANI chemistry after washing. The quinoid and benzenoid groups are labeled by * and δ, respectively.

Besides the as-deposited films, films were also soaked in THF after deposition and dried to investigate the effects of this post-deposition washing step. As mentioned, previous work on other oCVD polymers have shown improved electrochemical properties and stability with washing [17,31]. This has been attributed to the removal of oxidant and soluble oligomers from the films. The washed BC film, as seen in Figure 4a (0.8 sccm in the *F* series) or Figure 4b (700 mTorr in the *P* series), is typical PANI in the base form, with peaks at 1588, 1510, 1315, 1160, 1035, and 824 cm^−1^. The reduction in peak intensity around 1160 cm^−1^ after washing as compared to the as-deposited BC film suggests a transition from the salt to the base form of PANI (see XPS results below). Furthermore, peak shifts between the as-deposited and washed BC films also indicate that the film transitions from the doped salt form to the undoped base form. For instance, the 865 and 1160 cm^−1^ peaks of as-deposited BC have shifted by 30 and 54 cm^−1^, respectively, to higher wavenumbers in the washed film, and this indicates that the film has transitioned to the undoped base state [56]. Work by Trchová et al. [47] showed that only the base form of PANI contains a peak at ≈1315 cm^−1^ (the acid doped form of PANI shifts this peak lower by 10 cm^−1^), which is what is observed for the washed BC film. Similar to the as-deposited film, the peak at 825 cm^−1^ for the washed film is consistent with high molecular weight PANI due to para-di-substitution and suggests para-coupling of the chain units [48–49]. The oxidation state can be derived from the relative intensities of the 1588 quinoid and 1510 cm^−1^ benzenoid peaks, which for the washed film, gives a value of 0.87 and suggests that most of the washed BC polymer is in the emeraldine form. This makes oCVD a highly promising approach for a wide range of applications that can make use of the favorable properties of emeraldine PANI. The presence of emeraldine PANI is further supported by the green color of the washed PANI film (Figure 2b). In addition, previous oCVD PANI UV–vis measurements have also suggested the formation of emeraldine PANI [41].

The *F* series in Figure 4a shows washed films deposited under different oCVD operating conditions. As can be seen, similar to the FTIR spectra for the as-deposited films (Figure 3a), there is minimal influence of the oxidant flowrate down to 0.3 sccm. However, at the lowest oxidant flow rate of 0.15 sccm (washed F2 film), there is a lower peak intensity at 1588 cm^−1^, suggesting that the film is in the fully reduced leucoemeraldine state. Taking the ratio of the quinoid and benzenoid peak intensities leads to a ratio of 0.41, suggesting that the film is primarily composed of benzenoid groups with a low concentration of quinoid groups. This is expected because, with the much lower oxidant flowrate, there is probably insufficient oxidant available for oxidative polymerization and doping, thus leading to a lower oxidation state of the film. Further, with washing, the film becomes dedoped. For the *P* series (Figure 4b), again similar to the as-deposited counterparts, there does not seem to be a major effect of reactor pressure on film chemistry. As discussed above, we hypothesize that, in general, the oCVD PANI reaction is not pressure or concentration dependent based on the conditions studied. As for washing the lower substrate temperature films, LT-BC and LT-F1, it should be pointed out that both films completely dissolved in THF and therefore no FTIR of the washed films was possible. However, the ease of dissolution further supports our earlier conclusion that these conditions led to films that were primarily soluble oligomers.

### SEM and XPS of as-deposited and washed oCVD PANI films

**Figure 5:**
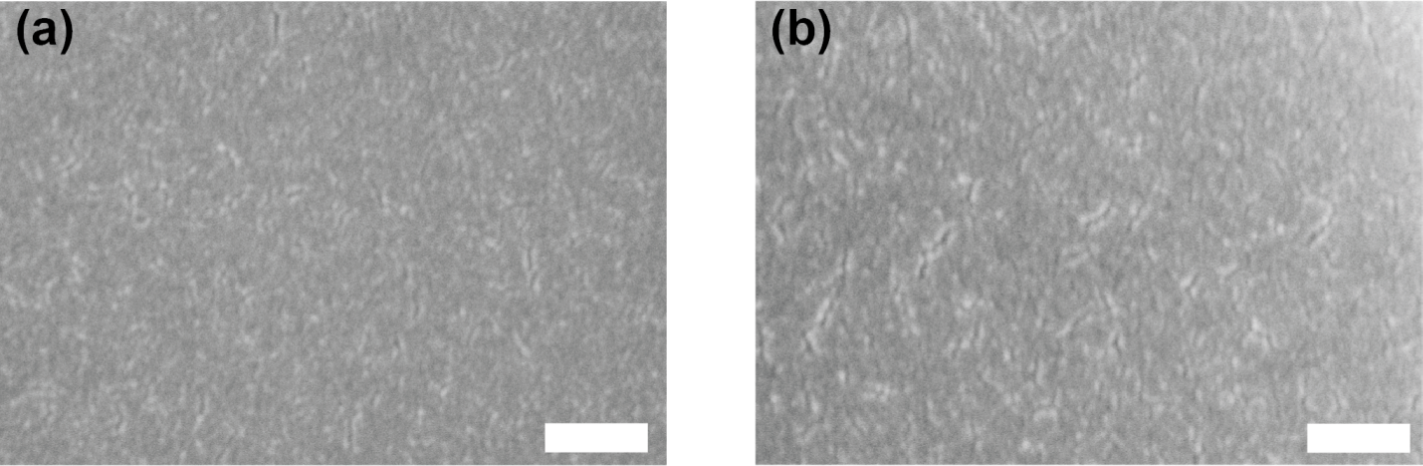
Top-down SEM of (a) as-deposited, and (b) THF-washed oCVD PANI films. Scale bar is 200 nm.

Given that the base case condition (Table 1) seems to have yielded the preferred emeraldine PANI state, further studies were carried out on both the as-deposited and washed BC films to detail their film chemistry and structure. As shown in the top-down SEM images presented in Figure 5, the film morphology did not visibly change after washing. Zoomed-out SEM images in the Supporting Information also show a uniform film morphology before and after washing (Figure S1, Supporting Information File 1). This qualitatively indicates that the BC film is stable and free of oligomers that would most likely alter film morphology if they were dissolved out of the film. Additionally, XPS was performed to understand more quantitatively the oxidation state and doping level of PANI before and after washing. The BC condition was chosen for analysis because our earlier FTIR findings indicated that high substrate temperature, pressure, and oxidant flowrate are favorable for depositing PANI by oCVD. To investigate the presence of the antimony pentachloride oxidant before and after washing, high resolution Cl2p and Sb3d core level XPS spectra were obtained (Figure S2, Supporting Information File 1). From these spectra, the amount of Cl and Sb in the as-deposited film was 6.9 and 11.59 atom %, while after washing, these values decreased to 1.24 and 0.34 atom %, representing a reduction of 82 and 97% reduction, respectively. This indicates that the doping level of the film significantly decreases after the washing process, and corroborates the FTIR finding that showed the BC film transition from the PANI salt to PANI base form when washed with THF.

**Figure 6:**
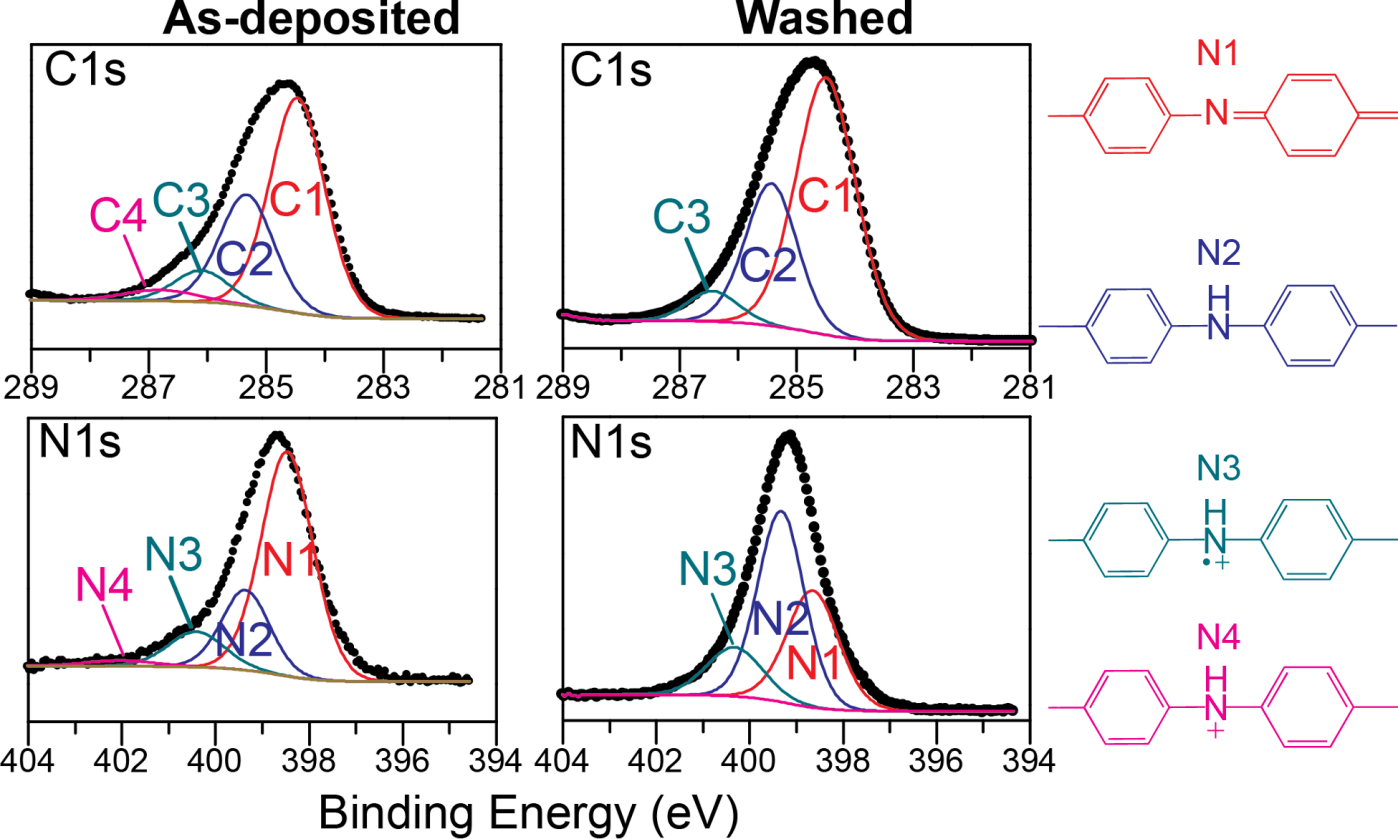
High-resolution C1s and N1s XPS spectra of as-deposited (left) and washed (right) oCVD PANI films. The resolved nitrogen bonding environments are assigned to non-equivalent nitrogen groups in PANI that are color-coordinated to the XPS spectra.

Further XPS was done to obtain the high resolution N1s XPS spectra of the BC film before and after washing, as shown in Figure 6. For the as-deposited film, the N1s spectrum can be resolved into four unique nitrogen bonding environments, see Table 2. The resolved peak positions and FWHM values are very similar to those reported for PANI [57–60]. The lowest binding energy state (N1) is a neutral imine (–N=) and comes from the base form of the emeraldine and pernigraniline structure of PANI (Figure 1). This peak is associated with the quinoid groups. The next state up (N2) is the neutral amine (–NH–), which is found in the base form of the leucoemeraldine and emeraldine states. It is associated with the benzenoid groups. The third higher energy nitrogen state (N3) is a cation radical amine state and comes most likely from the acid form of the emeraldine state. Finally, the fourth and highest-energy state (N4) can be attributed to a cation amine state, which comes from the salt form of PANI. From the resolved peak analysis, the relative amounts of N1, N2, N3, and N4 are 65.5, 21.5, 11.1, and 2.2 atom %, respectively (Table 2). In addition, by considering the intensity ratio of (N1 + N3 + N4)/N_total_, it is possible to determine the oxidation state of the as-deposited BC film, for example, a value of 0.5 indicates emeraldine PANI. For the as-deposited BC film, a ratio of 0.79 corresponds to a film that is ≈80% oxidized. This indicates that the film has a higher concentration of quinoid groups and therefore is highly oxidized. This validates the FTIR results which give the same conclusion. Upon washing, the resolved N1s spectrum shows the relative proportions of N1, N2, N3, and N4 are 34.2, 50.4, 15.4, and 0 atom %, respectively (Table 2). The most obvious change is the disappearance of N4. Since this aligns with the dramatic reduction in the antimony and chlorine dopant levels after washing and given N4 is a doped cation, this indicates that this state is formed as a result of the oxidant simultaneously enabling polymerization and doping of the growing PANI film. Similar to the as-deposited film, the oxidation state of the washed BC film can be determined by taking the ratio of (N1 + N3)/N_total_. For the washed BC film, this ratio is 0.49, which is very close to the theoretical value of 0.5 for PANI in the emeraldine form. This corroborates the FTIR results, which suggested the emeraldine state of the washed BC film.

**Table 2:** Resolved peak data from N1s and C1s XPS spectra of as-deposited and washed oCVD PANI films (BC condition).

	N1	N2	N3	N4	C1	C2	C3	C4

As-deposited								

Binding energy (eV)	398.5	399.4	400.4	402.0	284.5	285.3	286.1	286.8
Atomic %	65.2	21.5	11.1	2.2	58.0	29.6	8.5	3.9

Washed								

Binding energy (eV)	398.7	399.3	400.4	–	284.5	285.3	286.0	–
Atomic %	34.2	50.4	15.4	–	63.2	30.5	6.3	–

Likewise, XPS was carried out to obtain the high resolution C1s XPS spectra of as-deposited and washed BC films, as seen in Figure 6. The carbon signal for the as-deposited BC film can be resolved into four bonding environments, see Table 2. The lowest binding energy state (C1) corresponds to the C–C and C–H bonds, which for PANI, is due to the =CH– group [61]. The second lowest energy state (C2) can be assigned to neutral C–N bonds, which for PANI are those of N1 and N2, corresponding to the carbons bonded to neutral amine and imine nitrogens. The third, higher energy state (C3) is the carbon that is bound to the cation radical nitrogen (N3), while the highest energy state (C4) is given to a carbon bound to the cation nitrogen (N4). From Table 2, the fractions of C1, C2, C3, and C4 for the as-deposited BC film are 58.0, 29.6, 8.5, and 3.9%, respectively, while after washing, the proportions become 34.2, 50.4, 15.4, and 0%, respectively. Again, the loss of the C4 peak can be attributed to the removal of the dopant due to the washing process. Based on XPS work with electrodeposited PANI, Kumar and coworkers [60–61] stated that the resolved carbon peaks can be used to determine if PANI contains only para-coupling of the repeat unit. Specifically, if the intensity ratio of C1/(C2 + C3 + C4) is equal to 2, only para-coupling takes place within the ring. For our case here, the ratio is 1.4 and 1.7, respectively, for the as-deposited and washed BC films. The washed film is close to 2, which indicates primarily para-coupling. A ratio much lower than 2 suggests that there could be further ortho-coupling in addition to para-coupling of the aniline ring [60–61]. These structures seem to be removed with washing and could be related to less stable oligomer units.

## Conclusions

The oCVD process provides a viable approach for a one-step synthesis and deposition of PANI thin films using aniline monomer and antimony pentachloride oxidant. By carefully adjusting oCVD processing parameters, emeraldine PANI with its more desirable electrochemical properties can be formed. By varying the processing conditions, the oxidation level, doping concentration, and film chemistry, as determined by spectroscopy, could be controlled. Specifically, a high substrate temperature (90 °C) and a nearly equimolar ratio of monomer-to-oxidant feed flow rates that provides sufficient amount of oxidant is needed to produce PANI in the emeraldine state. This optimal oCVD condition has been shown to have superb electrochemical performance [41]. Lowering the substrate temperature to 25 °C or reducing the oxidant flowrate below 0.3 sccm leads to predominantly an oligomeric film. However, changing reactor pressure does not have any appreciable effect of the film chemistry. By washing oCVD PANI films with THF, which acts also as a dopant, soluble oligomer components can be removed effectively. This work, for the first time, identified synthesis conditions suitable for making PANI via oCVD, and revealed the influence of different processing parameters on film chemistry. The ability to use oCVD to produce emeraldine PANI is expected to open up new areas and applications, particularly in the field of electrochemical energy storage, which can benefit from the integration of thin PANI films without the issues of liquid processing.

## Supporting Information

File 1 Additional data.

## References

[1] Huynh W U, Dittmer J J, Alivisatos A P (2002). Science.

[2] Kim J Y, Lee K, Coates N E, Moses D, Nguyen T-Q, Dante M, Heeger A J (2007). Science.

[3] Li G, Zhu R, Yang Y (2012). Nat Photonics.

[4] Chen H-Y, Hou J, Zhang S, Liang Y, Yang G, Yang Y, Yu L, Wu Y, Li G (2009). Nat Photonics.

[5] Günes S, Neugebauer H, Sariciftci N S (2007). Chem Rev.

[6] Smolin Y Y, Nejati S, Bavarian M, Lee D, Lau K K S, Soroush M (2015). J Power Sources.

[7] Wu H, Yu G, Pan L, Liu N, McDowell M T, Bao Z, Cui Y (2013). Nat Commun.

[8] Zhang J, Zhao X S (2012). J Phys Chem C.

[9] Ramya R, Sivasubramanian R, Sangaranarayanan M V (2013). Electrochim Acta.

[10] Wang K, Wu H, Meng Y, Wei Z (2014). Small.

[11] Shi Y, Pan L, Liu B, Wang Y, Cui Y, Bao Z, Yu G (2014). J Mater Chem A.

[12] Lin H, Li L, Ren J, Cai Z, Qiu L, Yang Z, Peng H (2013). Sci Rep.

[13] Osada Y, De Rossi D E (2013). Polymer Sensors and Actuators.

[14] Ates M (2013). Mat Sci Eng C.

[15] Menard E, Meitl M A, Sun Y, Park J-U, Shir D J-L, Nam Y-S, Jeon S, Rogers J A (2007). Chem Rev.

[16] Grayson A C R, Shawgo R S, Johnson A M, Flynn N T, Li Y, Cima M J, Langer R (2004). Proc IEEE.

[17] Gleason K K (2015). CVD Polymers: Fabrication of Organic Surfaces and Devices.

[18] Huang W-S, Humphrey B D, MacDiarmid A G (1986). J Chem Soc, Faraday Trans 1.

[19] Zotti G, Cattarin S, Comisso N (1987). J Electroanal Chem Interfacial Electrochem.

[20] Andreatta A, Cao Y, Chiang J C, Heeger A J, Smith P (1988). Synth Met.

[21] Wang M, Wang X, Moni P, Liu A, Kim D H, Jo W J, Sojoudi H, Gleason K K (2016). Adv Mater.

[22] Eason R (2007). Pulsed Laser Deposition of Thin Films: Applications-Led Growth of Functional Materials.

[23] Mercado A L, Allmond C E, Hoekstra J G, Fitz-Gerald J M (2005). Appl Phys A.

[24] Chrisey D B, Piqué A, McGill R A, Horwitz J S, Ringeisen B R, Bubb D M, Wu P K (2003). Chem Rev.

[25] Hansen S G, Robitaille T E (1988). Appl Phys Lett.

[26] Johnson S, Park H, Haglund R (2007). Appl Surf Sci.

[27] Im S G, Kusters D, Choi W, Baxamusa S H, van de Sanden M C M, Gleason K K (2008). ACS Nano.

[28] Jo W J, Nelson J T, Chang S, Bulović V, Gradečak S, Strano M S, Gleason K K (2016). Adv Mater.

[29] Chen N, Kovacik P, Howden R M, Wang X, Lee S, Gleason K K (2015). Adv Energy Mater.

[30] Barr M C, Rowehl J A, Lunt R R, Xu J, Wang A, Boyce C M, Im S G, Bulović V, Gleason K K (2011). Adv Mater.

[31] Nejati S, Lau K K S (2011). Langmuir.

[32] Nejati S, Minford T E, Smolin Y Y, Lau K K S (2014). ACS Nano.

[33] Nejati S, Patel A, Wallowitch G R, Lau K K (2015). Nanosci Nanotechnol Lett.

[34] Snook G A, Kao P, Best A S (2011). J Power Sources.

[35] Bhadra S, Khastgir D, Singha N K, Lee J H (2009). Prog Polym Sci.

[36] Chiang J-C, MacDiarmid A G (1986). Synth Met.

[37] Lee S, Paine D C, Gleason K K (2014). Adv Funct Mater.

[38] Howden R M, McVay E D, Gleason K K (2013). J Mater Chem A.

[39] Chelawat H, Vaddiraju S, Gleason K (2010). Chem Mater.

[40] Nardes A M, Kemerink M, de Kok M M, Vinken E, Maturova K, Janssen R A J (2008). Org Electron.

[41] Smolin Y Y, Van Aken K L, Boota M, Soroush M, Gogotsi Y, Lau K K S (2017). Adv Mater Interfaces.

[42] Genies E M, Tsintavis C (1985). J Electroanal Chem Interfacial Electrochem.

[43] Hatchett D W, Josowicz M, Janata J (1999). J Phys Chem B.

[44] Neugebauer H, Neckel A, Sariciftci N S, Kuzmany H (1989). Synth Met.

[45] Patil D S, Shaikh J S, Dalavi D S, Kalagi S S, Patil P S (2011). Mater Chem Phys.

[46] Šeděnková I, Trchová M, Blinova N V, Stejskal J (2006). Thin Solid Films.

[47] Trchová M, Šeděnková I, Tobolková E, Stejskal J (2004). Polym Degrad Stab.

[48] Stejskal J, Trchová M (2012). Polym Int.

[49] Zaharias G A, Shi H H, Bent S F (2006). Thin Solid Films.

[50] Ping Z (1992). J Chem Soc, Faraday Trans.

[51] Abdiryim T, Xiao-Gang Z, Jamal R (2005). Mater Chem Phys.

[52] Trchová M, Stejskal J (2011). Pure Appl Chem.

[53] Cao Y, Li S, Xue Z, Guo D (1986). Synth Met.

[54] Im S G, Gleason K K, Olivetti E A (2007). Appl Phys Lett.

[55] Lock J P, Im S G, Gleason K K (2006). Macromolecules.

[56] Li J, Tang X, Li H, Yan Y, Zhang Q (2010). Synth Met.

[57] Tan K L, Tan B T G, Kang E T, Neoh K G (1989). Phys Rev B.

[58] Golczak S, Kanciurzewska A, Fahlman M, Langer K, Langer J J (2008). Solid State Ionics.

[59] Zeng X-R, Ko T-M (1998). Polymer.

[60] Kumar S N, Gaillard F, Bouyssoux G, Sartre A (1990). Synth Met.

[61] Kumar S, Bouyssoux G, Gaillard F (1990). Surf Interface Anal.

