# Supplementary material for: Oxidative chemical vapor deposition of polyaniline thin films
Source: Beilstein J Nanotechnol. 2017 Jun 16;8:1266–76. doi: 10.3762/bjnano.8.128 (PMC5480348; doi:10.3762/bjnano.8.128)
Supplement: File 1 — Additional data. [file Beilstein_J_Nanotechnol-08-1266-s001.pdf]

# Supporting Information

# for

# Oxidative chemical vapor deposition of polyaniline thin films

Yuriy Y. Smolin[‡], Masoud Soroush[‡] and Kenneth K. S. Lau[*,‡]

Address: Department of Chemical and Biological Engineering, Drexel University, Philadelphia, PA 19104, USA

Email: Kenneth K. S. Lau - klau@drexel.edu

* Corresponding author

‡ Equal contributors

# Additional data

# Scanning Electron Microscopy

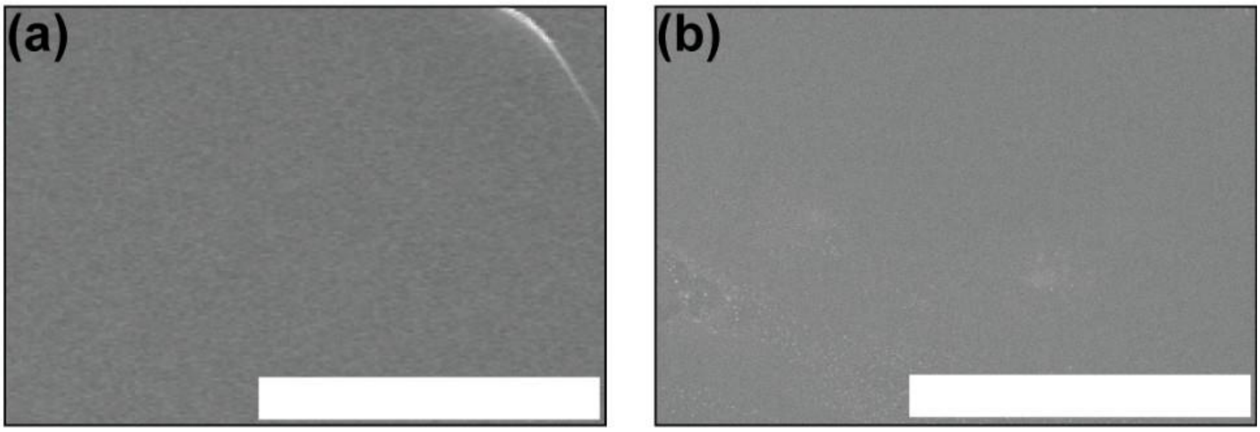


**Figure S1:** Top-down SEM of (a) as-deposited, and (b) THF-washed oCVD PANI films at lower magnification. Scale bar is 10 µm.

# Fourier Transform Infrared Spectroscopy

**Table S1:** Major FTIR peak positions and assignments for oCVD PANI.

| Peak Position ($cm^{-1}$) | Peak Assignment | Reference (in main paper) |
|---|---|---|
| 3304 | NH stretch | [44-46] |
| 3100–3000 | CH stretch | [44-46] |
| 1168–1160 | –$NH^{+}$= stretch / CH vibration | [46,47] |
| 865–821 | CH vibration (out-of-plane) | [47-49] |
| 1588–1577 | Quinoid ring stretch | [50,51] |
| 1510–1490 | Benzenoid ring stretch | [50,51] |
| 1382 | CN stretch of quinoid ring | [50] |
| 1635 | NH scissoring vibration of aromatic amines | [52] |
| 749 | CH out-of-plane bending | [48] |
| 688 | CH out-of-plane ring deformation | [48] |

## X-ray Photoelectron Spectroscopy

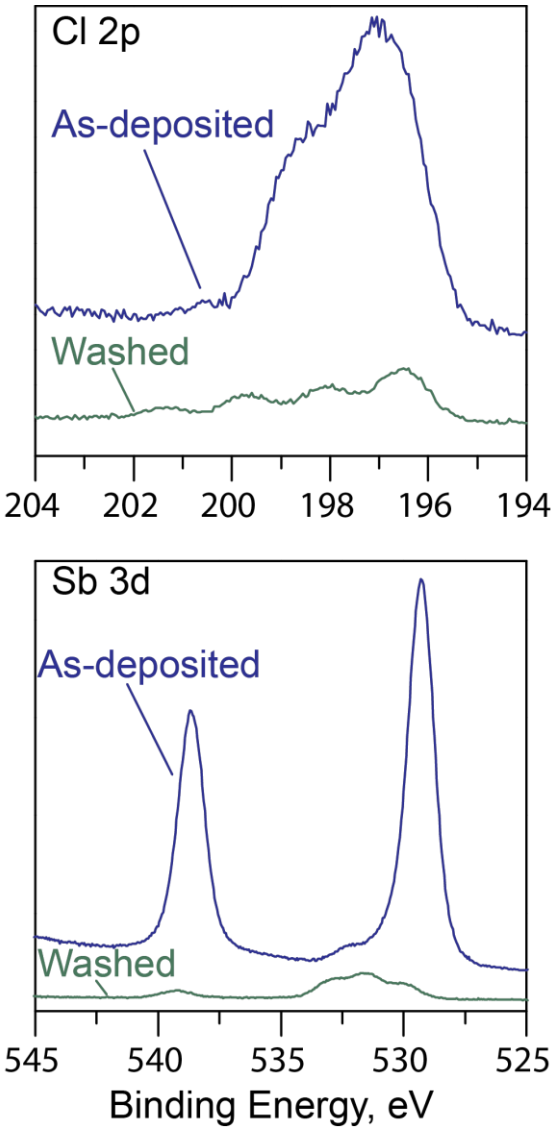


**Figure S2:** High-resolution Cl2p (top) and Sb3d (bottom) XPS spectra of as-deposited (blue) and washed (green) oCVD PANI films.
